# Allied health professionals’ experiences and views towards improving musculoskeletal services in the UK for patients with musculoskeletal and co-existing mental health conditions: a qualitative study

**DOI:** 10.1186/s12891-023-06878-w

**Published:** 2024-03-07

**Authors:** Rokhsaneh Tehrany, Dana Maki, Maria J C Teixeira, Tanya Chumak, Christine Hoerz

**Affiliations:** 1 Department of Health Sciences, College of Health, Medicine and Life Sciences, Brunel University, London, UK; 2 Alanzoor Physiotherapy & Rehabilitation Complex, Manama, Kingdom of Bahrain; 3 Therapies Department, Royal National Orthopaedic Hospital NHS Trust, London, UK; 4 Department of Orthopaedic and Musculoskeletal Science, University College London, London, UK; 5 Nursing Research Department, Royal National Orthopaedic Hospital NHS Trust, London, UK; 6 London South Bank University, London, UK; 7 Nuffield Health Oxford, The Manor Hospital, Oxford, UK

**Keywords:** Anxiety, Allied Health Professionals, Depression, Mental Health, Musculoskeletal, Perception, Physiotherapist, Occupational therapist, Training

## Abstract

**Background:**

Interplay between physical and mental health (MH) is widely recognised amongst patients with Musculoskeletal and co-existing MH conditions. Evidence suggests that psychological interventions improve outcomes and satisfaction in patients with physical conditions, however current healthcare models continue to separate physical and mental health care, as health services are fragmented. If the delivery of MH support could be facilitated by Allied Health Professionals (AHPs), such as physiotherapists and occupational therapists (OTs), this could be an effective, low-cost way to achieve routine integration. This study aimed to explore the experiences of UK physiotherapists and OTs working with patients with MSK and co-existing MH conditions and to understand views on improving MSK services.

**Methods:**

This was an exploratory-descriptive qualitative study using semi-structured interviews. Participants were recruited via social media and professional organisations using convenience sampling. Participants included registered UK physiotherapists or OTs within MSK settings who managed patients with MH conditions. Inductive thematic analysis was used, where single and double-level coding, single counting and inclusion of divergent cases were conducted to enhance methodological rigour.

**Results:**

Three overarching themes were identified. Overarching theme one referred to *openness to provide MH support*, with *scope of practice* and *lack of confidence* as themes. Overarching theme two described *challenges*, incorporating *mental health stigma*, the *clinical environment*, and *limited experience*. The overarching theme referring to *training*, identified the need for *further training* and *strategies to implement* as themes.

**Conclusion:**

Many challenges to achieving optimal integration of physical and mental health care exist within MSK services. These challenges go beyond the need for additional training and knowledge acquisition and include departmental readiness such as funding, diary management, and supervision by senior colleagues/or psychologists. These need consideration in parallel to match the evolving needs of the MSK population.

**Supplementary Information:**

The online version contains supplementary material available at 10.1186/s12891-023-06878-w.

## Background

In the United Kingdom (UK), around a third of the population (20.3 million people) lives with a Musculoskeletal (MSK) condition involving the joints, muscles and bones, such as arthritis and low back pain [1]. Musculoskeletal conditions lead to a range of life altering symptoms, such as persistent pain, stiffness, and fatigue [2, 3]. These symptoms infringe on quality of life due to the wide-ranging impacts on independence and the ability to maintain social relationships, or occupational roles [4]. Accordingly, the association between MSK and mental health conditions (such as anxiety and depression) has become increasingly recognised [3, 5, 6].

One in four adults reportedly experience at least one diagnosable mental health condition in the UK [7], where anxiety and depression involving ongoing episodes of relapse and remission represent the most commonly diagnosed psychological conditions amongst adults [8]. People with long-term MSK conditions are likely to report feeling anxious or depressed when compared to healthy individuals [9, 10] and there is evidence to support the bidirectional relationship. For example, anxiety and depression might heighten sensation of pain associated with MSK conditions, while persistent pain might worsen mental health [9, 11–13]. In 2021, national statistics indicated the prevalence of self-reported mental health conditions in the UK was higher amongst people with MSK conditions, when compared to those without (odds ratio 1.4) [14]. For patients with both physical and mental health conditions, higher rates of morbidity, healthcare utilisation and poorer quality of life have been observed [15]. Interplay between MSK conditions and anxiety and depression, therefore, represents a major public health issue [5], which highlights the need to optimise the management of patients who present with psychological symptoms within the MSK setting.

There is evidence that a biopsychosocial approach involving psychological interventions, such as Cognitive Behavioural Therapy (CBT) and mindfulness, result in positive health outcomes, improved functioning and reduced healthcare utilisation for patients with MSK conditions and co-existing anxiety and depression [16, 17]. Furthermore, there is mounting interest towards evaluating the efficacy of psychological interventions delivered by non-psychologists [18, 19]. A systematic review and meta-analysis indicated that physiotherapist delivered psychological interventions in combination with physiotherapy is more effective than physiotherapy alone for improving pain and disability outcomes. While the effect sizes were small and the quality of evidence ranged from high to low, small to medium effect sizes were reported for some psychological outcomes and all effects favoured the intervention [16]. These findings highlight opportunities for non-psychologists (such as Allied Health Professionals (AHPs)) to integrate the management of physical and mental health care within an MSK setting.

Allied Health Professionals, such as physiotherapists and occupational therapists (OTs), provide assessment, diagnosis and first-line management of MSK conditions, hence the feasibility of integrating mental and physical healthcare will require their acceptability and active engagement [16]. Driver et al. (2016) conducted a systematic review evaluating the knowledge, behaviours and attitudes of physiotherapists towards the use of psychological interventions. The findings indicated that although physiotherapists are aware of psychological interventions and have positive attitudes towards their use, barriers to implementation also exist, including lack of knowledge, role clarity and time constraints [20]. Other studies involving physiotherapists have also reported similar findings [21, 22], however considerable heterogeneity amongst these studies exist, which limits the generalisability. For example, a number of these studies have either focussed on the views of physiotherapists who managed elite athletes [23, 24], included findings from surveys [21, 25], or involved qualitative interviews that were conducted in other countries, such as the US or Australia [26–28]. Furthermore, while much attention has focused on understanding the views of physiotherapists towards their perceived role in managing co-existing psychological conditions, the full picture surrounding the perceived barriers and facilitators, as well as views on how to improve MSK services for patients with co-existing mental health conditions is less clear.

Fewer studies have explored the perceptions of OTs [29], even though mental health is a taught component within the OT undergraduate curriculum, and OTs have experience with delivering a range of psychological interventions, such as psychoeducation, time use/occupational balance and skills habit development [30, 31]. In light of the increasing role overlap between physiotherapists and OTs, the therapeutic benefits associated by the multidisciplinary approach [5, 32–34], and the growing body of evidence suggesting the potential effectiveness of the biopsychosocial model in the MSK setting [5], there is a need to consider the perspectives of both professions to gain a clearer picture about the barriers and facilitators to integrating the management of physical and mental health to enhance MSK services, as well as views on anticipated support needs to improve care.

The aim of this study was to explore the experiences of AHPs in the UK, particularly physiotherapists and OTs, working within the MSK setting, with patients with co-existing signs and symptoms of anxiety and depression. These symptoms are here on referred to as ‘co-existing mental health (MH) conditions’. A secondary aim was to understand their views on changes and support required to improve MSK services for this patient population.

## Methods

### Design

A qualitative, experiential-descriptive study was conducted to explore the experiences of physiotherapists and OTs working within an MSK setting with patients with co-existing MH conditions, and to understand their views on the further support needed to improve patient care [35].

Ethical approval was granted from Brunel University London, College of Health, Medicine and Life Sciences Research Ethics Committee (Reference: 33,127-MHR-Feb/2022-37827-3).

### Participants

The aim was to recruit a maximum of 12 physiotherapists or OTs registered and working within the UK, or stop at saturation. A maximum sample of 12 participants compared to larger samples, was set following recommendations by the review Guest, Bunce & Johnson (2006), to allow in-depth meaningful data collection using semi-structured interviews [36]. Data collection stopped once data saturation was reached at 10 participants, when new codes or themes no longer emerged, and when the data repeated what was previously expressed [37], through continuous analysis and reflection [35].

Potential participants were considered for inclusion if they were physiotherapists or OTs working within UK MSK setting, who managed patients with co-existing anxiety or depression. There were no limits on the length of experience within those settings to allow for a variety of experiences to be included. Participants who exclusively worked in paediatric or spinal cord injury (SCI) settings were excluded. A decision to exclude AHPs working within SCI was taken on the basis that the biopsychosocial approach is already widely implemented amongst this population [38], hence their experiences might differ from those exclusively working within an MSK setting.

### Recruitment

Convenience sampling, involving recruitment through social media (Twitter) and Interactive Chartered Society of Physiotherapy (iCSP), commenced from January to March 2022. A link was shared to allow potential participants to read the Participant Information Sheet (PIS), undergo a self-assessment against pre-determined inclusion and exclusion criteria, before giving electronic Informed Consent.

Participants’ characteristics were collected through an online questionnaire, this included type of work (NHS, or non-NHS), years since qualification, and number of years in MSK practice.

### Data collection

A semi-structured interview guide (see Appendix 1) was formulated following a review of the literature [16, 18, 20, 30] and in-depth discussions between a qualitative researcher (who is a specialist MSK physiotherapist), a senior lecturer in physiotherapy, and a physiotherapy researcher (who is currently supporting a programme of work exploring the efficacy of interventions for optimising the integration of MSK and mental health management). This guide included three semi-structured questions, with a pre-determined list of prompts to ensure consistency between the interviewers.

Interviews commenced between February and March 2022, by two student researchers as part of their fulfilment for a dissertation for an MSc (pre-registration) in Physiotherapy at a UK based University. Student researchers were trained to conduct in-depth, one-to-one qualitative interviews. The questions were then piloted with other MSc candidates who had an interest in MSK specialities with co-existing MH conditions. Reflexive journals and field notes were maintained throughout the interview phase.

### Data analysis

Inductive thematic analysis (ITA) was used to analyse the transcripts, with the aim to explore data based on the pragmatic paradigm as it was aimed to understand the reality in practice having a focus on addressing ‘real-world’ problems (Hothershall, 2019). The goal is to change reality by taking into consideration the findings. NVivo 12 was used to analyse all qualitative data.

The interviews were conducted virtually and transcribed automatically by Microsoft Teams. Student researchers anonymised and reviewed the transcripts against the audio recordings to check for errors and subsequently rectified any inconsistencies. Therefore, level one coding involved the review and familiarisation of the transcripts, as well as presentation of the preliminary list of codes [35]. Level two coding involved the removal of redundancies and agreements on codes following discussions and coding by the same two students and two experienced qualitative researchers (DM and TJ) DM and TJ only accessed anonymised transcripts.

The continuous analysis, which included level one and two coding and cross-referencing with the wider research team, was used to determine data saturation and when to cease recruitment. Disagreements were also resolved during this process to enhance the validity of the results [39]. The practice of level one and two coding, and continuous discussion enabled the researchers to actively interpret the data by identifying and developing themes [40]. This helped to reduce uncertainty with data saturation [41], enabled the team to practise reflexivity and share any notes from reflexive journals maintained during interviews or analysis [42]. Single counting and inclusion of divergent cases were applied to show the strength of the evidence from the available qualitative data and to further validate the results [43]. The final framework of overarching themes, themes and sub-themes were discussed with the research team [42].

### Reflexivity statement

Reflexivity was practised by the entire research team, which incorporated the senior authors’ research experience with the clinical experience of all authors through formal meetings to discuss the data. DM and JT have had experience of using thematic analysis for over 10 years. DM, JT, and RT are both clinicians and academics who have been working with patients with chronic musculoskeletal conditions for a combined period over 20 years and are also involved in several research projects surrounding mental health and MSK conditions. TC and CH are both MSc (pre-reg) physiotherapy students near the completion of their degree. TC has had experience within MSK practice as a student, and CH has experience within MSK practice that included patients with co-existing MH conditions. The research team strived to remain aware of their own positionings by reflecting on all of their experiences, and to improve the rigour of data analysis.

## Results

### Participants

Ten participants were recruited in total (seven physiotherapists and three OTs). All participants were female and working within the NHS except two physiotherapists who worked in non-NHS settings. Physiotherapists and OTs had an average of 22 years and 13.05 years since qualification respectively. Physiotherapists had an average of 17 years of experience working with patients with MSK conditions, whilst OTs had 13.05 years (Table 1).

**Table 1:** Participant characteristics

Participant	Professional Experience*	Band Level	MSK Experience*	Health System	Clinical Speciality
OT 1	< 1	5	< 1	NHS	Rotational OT
OT 2	7	6	7	NHS	Outpatients
OT 3	32	7	32	NHS	Chronic pain and MSK services
Physio 1	35	7	25	Non- NHS	Private MSK, part-time
Physio 2	14	8b	12	NHS	Community and orthopaedics in secondary care
Physio 3	34	7	32	NHS	Occupational health
Physio 4	17	7	17	NHS	Acute elderly, community, and outpatient rehab
Physio 5	24	8b	18	NHS	MSK outpatients
Physio 6	22	8a	15	Non- NHS	Private MSK
Physio 7	8	7	6	NHS	MSK outpatients

Key:

MSK: musculoskeletal practice

*professional and MSK experience is measured in years

### Findings

**Fig. 1:**
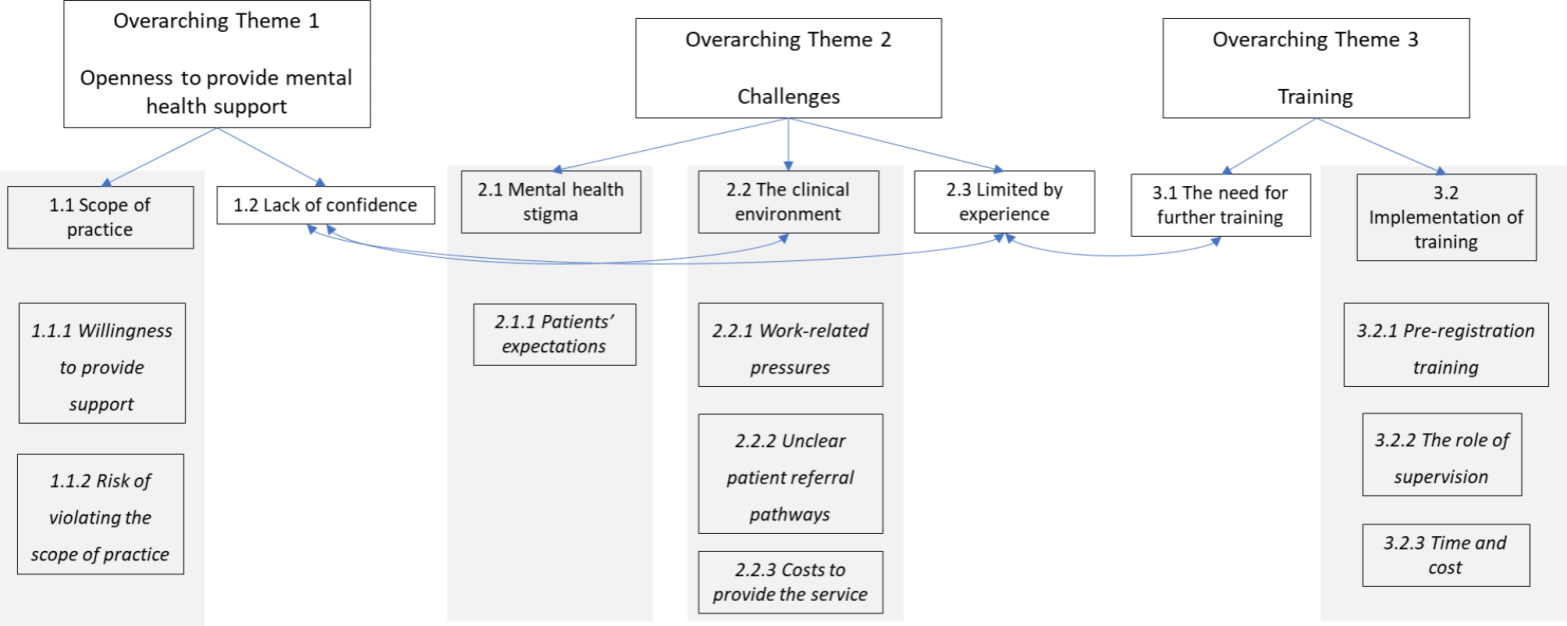
The overarching themes, themes and interlinked sub-themes

Three overarching themes which related to supporting patients with co-existing MH conditions were identified following the ITA, namely [1] openness to provide MH support [2], challenges and [3] training needs to improve patient’s experience. The overarching themes were underpinned by themes, and relevant sub-themes, see Table 2. Themes were interlinked through some sub-themes, (see Fig. 1).

**Table 2:** Overarching themes, themes and subthemes

	Overarching Themes	Themes	Sub-themes
1	Openness to provide MH support	Scope of practice	*Willingness to provide support* *Risk of violating the scope of practice*
		Lack of confidence	
2	Challenges	Mental health stigma	*Patient’s expectations*
		The clinical environment	*Work-related pressures* *Unclear patient referral pathways* *Costs to provide the service*
		Limited by experience	
3	Training	The need for further training	
		Implementation of training	*Pre-registration training* *The role of supervision* *Time and cost*

All participants recognised that mental health conditions are underdiagnosed and poorly managed during the rehabilitation pathway and a biopsychological approach is viewed as favourable in contrast to managing MSK conditions in isolation.

*“Just with the whole ordinary population, there’s…depression, anxiety and stuff going on, as well as actual undiagnosed and diagnosed mental health issues.”* **Physio 2**

*“There is a significant number of people that we see, that have… significant mental health problems that are barriers to their rehab…”* **Physio 4**

## Overarching theme 1: openness to provide mental health support

All but one participant, expressed an openness to support patients presenting with mental health conditions, such as anxiety and depression. Two main themes are presented here: scope of practice (willingness to provide support and risk of violating the scope as sub-themes), and lack of confidence.

### Scope of practice

Most participants agreed that it was within the role of the physiotherapist or OT to look after the ‘whole person’, and some aspects of MH support were within their scope of practice.

*“I think it fits really nicely in the role of OT. I think we’re really well set up to look after the whole person.”* **OT 2**

*“I think it would be a positive impact…as we are already holistic in our approach anyway, having extra tools in our toolkit would be useful. And in order to sort of just progress that little bit further and then like I said, breakdown, any barriers to rehab.“* **Physio 7**

*“I think it increases our efficacy. That’s the top or bottom of it, is it? It makes our practice more effective.“* **Physio 4**

#### Willingness to provide support

Participants were willing to provide mental health support to some extent, and shared potential solutions to improve their confidence. For example, using available guidelines or protocols, training to identify MH conditions through signs and symptoms, and/or identifying suitable mentors, such as senior practitioners or psychologists.

*“And then it would be nice to have I guess sort of mental health champions, you know, somebody that you could turn to within the trust, somebody that might kind of take that forward a little bit more and then you know, like you do with safeguarding.”* **Physio 5**

Participants felt that validation, empathy, and encouragement were within their scope of practice. Relaxation and breathing techniques were appropriate treatment techniques. Patients presenting with fear-avoidance behaviours, or pain-related anxiety were managed within their scope of practice.

*“I think if we could make it appropriate so that we weren’t trying to be counsellors, we’re trying to do it so that it improves their physical health… so we’re not digressing from our profession.*” **Physio 1**

#### Risk of violating the scope of practice

Most of the participants across both professions disclosed their concerns about the risk of addressing symptoms of anxiety and depression in the absence of oversight from other professionals, such as psychologists.

*“And sometimes you might not want to cross boundaries by thinking you’re going into another discipline you know.”* **OT 1**

*“… you have to be careful, I suppose that you don’t sort of feel like you’re going into an area that you’re not really qualified in.”* **Physio 5**

*“I think it’s generalised anxiety and depression, depression, anxiety disorder, and that’s what’s most often diagnosed either before they come to us or, you know, as therapists we kind of make a tentative sort of diagnosis ourselves, although, you know it clearly outside of our scope of practice.”* **Physio 4**

### Lack of confidence

All participants except one reported lack of confidence towards providing MH support for anxiety and depression, which is strongly linked to the clinical environment they practiced within (see Overarching Theme 2: Challenges).

*“… our focus is still on the biomedical bit of it which is important […] we think that we are following the psychosocial bit […]. We’re not, we don’t have the skills. We don’t have the training to do it.”* **Physio 6**

*“I think that sometimes you feel like you’re not fully equipped to give some, you know, expert advice in that area.”* **OT 1**

One participant highlighted that AHPs with anxiety or depression might not have the emotional capacity to address psychological conditions with patients, regardless of their level of knowledge/experience.

## Overarching theme 2: challenges

All participants contributed to these themes: their experiences of challenges previously encountered while managing patients who had both an MSK and a co-existing MH condition. Three themes were highlighted in this overarching theme: mental health stigma, the clinical environment and limited experience.

### Mental health stigma

Depression, anxiety, and other psychiatric and mental health conditions continue to be omitted from conversations between patients and AHPs due to the stigma that still exists.

*“I think they forget to talk about it because in general, mental health has a big stigma or people are just kinds of shy away from it because you don’t want to say the wrong thing or you just feel like, […] you don’t feel equipped to even speak on the matter.”* **OT1**

*“I think there’s still some stigma out there, to be absolutely honest. It’s how you receive it, so people [AHPs] have got to want to receive it.”* **Physio 3**

#### Patients’ expectations

Participants shared their views on what patients might expect. A few participants reported that patients do not expect, and hence might not engage with MH support from AHPs, as they identify these AHPs as professionals who manage biomechanical symptoms (such as pain, stiffness and weakness), and not psychological symptoms. Participants suggested engaging patients with goal setting and reflections on values. They could use these motivational techniques to underpin physical goal attainment, which was more closely aligned with patients’ expectations of them.

*“…And I think it’s quite a skill to talk to people about their condition and their mental health without them feeling that you’re dismissing or just saying that it, you know, it’s all in your mind.”* **Physio 4**

*“I think some of the challenges, people don’t expect you to be doing this with you because it is, some of it is almost a bit of talking therapy. So, what I’ve found particularly helpful is talking about values. Because some of the acceptance commitment stuff is to do with committed action and values which is what from a physiotherapy point of view, we are probably reasonably familiar with.”* **Physio 6**

Nevertheless, participants anticipated that patients would embrace MH support from AHPs.

*“I think that they would actually react pretty well, because as soon as you meet a patient you initially want to build that therapeutic rapport with him. So, if you’ve already started that and then you realise, oh, there’s this mental health diagnosis there then I think that they would take it on very well because they already have that sense of interaction with you.” ***OT 1**

Participants indicated that further training would support them to identify key signs associated with MH conditions, which could trigger the need to investigate/refer thereafter. Most participants said that training would improve their confidence. Training can help them identify appropriate windows to discuss MH. The optimal timing of these conversations might help meet patients’ expectations and therefore enhance the management of both physical and psychological health.

*“I suppose, trying to engage somebody as well. Yeah, I think it’s really difficult. It’s not what we’re trained in, but is there a way to sort of, I wouldn’t say fast track into that understanding, but I think we’ve got to have a better understanding of it because we’re just doing so many people a disservice, really, aren’t we?”* **Physio 5**

*“It would be nice if some people had psychological support and physio at the same time and that doesn’t work I guess that’s my idea of trying to combine them both and it’s a challenge and it’s difficult and it takes I think the confidence to kind of discuss that with somebody, unless… from a patient perspective, they [are] expecting that”.* **Physio 6**

Several participants reported that understanding family relationships, the patients’ environment, and perhaps even family involvement as critical for successful patient engagement.

*“So that’s a thing you have to manage the person and then if you work in other environments, you’re also managing their family. It’s not just the person. It’s their families’ mental health problems that are influencing the way your patient is. And so, you know, it can just become really complex.”* **Physio 2**

### The clinical environment

Most participants reported that the feasibility of providing support to MH conditions was influenced by the work setting AHPs practiced in.

*“…the expectations of the mental health OT are very different from the expectations of the Community OT, which is very different from the expectations with inpatient OT.”* **OT 2**

#### Work related pressures

Participants stated that work pressures to see many patients and achieve targets influenced their ability to engage with patients who had additional MH needs on occasions.

The majority of participants identified large caseloads as a major barrier to providing MH support, as patients with co-existing anxiety and depression would benefit from longer therapy sessions, which isn’t feasible within the set up of current MSK pathways.

*“… so we haven’t got time to address it, but the other part is you have to because otherwise, they’re not going to get better…”* **Physio 1**

Participants highlighted that work-related pressures reduced their ability to manage complex patient needs regardless of their level of confidence or experience with managing psychological conditions.

*“I can deal with it in small amounts, but not in large amounts, because it does take a lot from clinicians to properly try and manage it.”* **Physio 2**

Participants reported that patients with co-existing MH conditions were more complex and could have a greater impact on AHP’s emotional and physical wellbeing when compared to patients with less complex needs.

*“You have those patients that you know, [who] are draining and mental health patients often are quite draining, to be honest with you ‘cause they need more, they need more support and motivation.”* **Physio 3**

*“you know, it’s very, very difficult to deal with… an hour-long appointment when somebody is., really distressed and tearful and, you know, suicidal sometimes. It doesn’t happen very often, but it does happen. And so sometimes we do have to… take the clinicians aside and kind of say it’s OK to be upset about this. It’s OK to be affected by this. It’s normal… I think they that you feel like once you put this uniform…. you know [like] it’s Teflon* " **Physio 4**

*“I found it massively overwhelming. And I wonder… if that could have been a contributing factor to me leaving the NHS… not just depressed people, but I think I found working with people and all their emotions all day just too much actually.”* **Physio 1**

#### Unclear referral pathways

Most participants raised concerns about their departmental readiness to support patients presenting with MH symptoms, even if they had already received appropriate MH training. While participants depended on onward referrals to support patients with unmet MH needs, seven participants felt that referral pathways were unclear and limited guidance was available within their clinical environment.

*“Signposts, signposts [are] a big one because I think that sometimes you feel like you’re not fully equipped to give some expert advice in that area.”* **OT 1**

*“So I think that identifying what the patients’ need earlier and having better resources for that would definitely be better, yeah.”* **Physio 2**

The majority of participants expressed that limited psychological services within their trust contributed to the challenges associated with managing patients presenting with physical and psychological needs.

*“[…]there’s a massive shortage in clinical psychologists… so I think that’s the problem.”* **Physio 2**

*“[I] think the disappointing thing is it’s quite difficult to get a psychological [assessment] and enough treatment, because I think sometimes people are offered an assessment and then their treatment doesn’t start till later on or it’s part of a group or it is it’s just a few sessions. And so sometimes it’s just not enough really, which is a bit disappointing.”* **Physio 6**

#### Costs to provide the service

Participants reported NHS services were underfunded, and perceived this to be the main contributor to the limited psychological resources available to them. This sub-theme also relates to overarching theme 3: Training (time and cost). This was described by one participant.

*“It wasn’t provided by our service, but we sort of linked up with them. And I think their service was underfunded.*” **Physio 5**

### Limited by experience

Most participants expressed that limited experience contributed to their diminished confidence. This theme interlinks with lack of confidence (overarching theme 1) and training needs (overarching theme 2).

*“I think there probably is a lack of confidence and there’s a bit again, it’s a fear that people are worried that if they ask questions about mental health and that kind of opened up a can of worms and they won’t be qualified and they don’t know what to do and how to deal with it.”* **Physio 1**

*“I’ve learned a blend over the years. I can use bits of what I know to sort of help, but let’s say I said I don’t. I feel like there’s a block to where I can get to with patients.”* **Physio 7**

## Overarching theme 3: training

This overarching theme is interlinked with the previous two themes, as the need for further training was linked to *limited by experience*, and *lack of confidence*. Training amongst participants was described as any theoretical or practical course or model that may improve skills to provide better support to patients with MH conditions. Two main themes were disclosed: the need for further training, and implementation of training.

### The need for further training

All participants expressed the need for further training to identify and manage MH conditions, such as anxiety and depression. Furthermore, they recognised the need to conduct initial assessments and early interventions to support patients. Five participants had training outside of their mandatory training, mainly in counselling and motivational interviewing.

*“And what I think would be really helpful would be, “yes, this depression elicits anxiety, or this is ADHD or this psychosis” or whatever it might be, “Which impacts this way and the way that we manage that is with this treatment.” I think that’s what we are missing.”* **OT 3**

“*So having some sort of formal, even just recognition that you are treating their depression. As well as some guidance as to how to treat their depression would be helpful in the future.*” **Physio 4**

“ *I guess it [training] would have to be something which is applicable to us. … I think that’ll be really good to have an overview of all of those [MH conditions], and those that are that are managed medically… the medications which they are on, which they take, can that affect them in a physical way?” ***Physio 2**

Further training and learning will eventually enhance the confidence of AHPs.

*“I do feel more confident, and it has changed my approach, but because it’s still new to me, I don’t feel that I’m very slick with applying it and I’m not.”* **Physio 6**

### Implementation of training

Participants shared suggestions on how training in psychological skills could be implemented.

#### Pre-registration training

Half of the participants reported the importance of psychological training in their pre-registration curriculum. It was suggested that basics should be taught pre-registration, and further training was to be provided when necessary, as AHPs became more experienced post-qualification.

*[…]“… at the undergrad level, what we learn is just the basics in everything, the basics of how the body works and, you know, the basics of mental health as well.”* **Physio 5**

*“I think it needs to come together. We can’t just have like an anatomy module and like a physiology module and like a mental health module, it needs to be like a health module. Because it impacts everything. And I think that would be… key… for so many people to understand is that it’s not anatomy and physiology and mental health. It’s the whole system feeding into itself together. And that’s how the person is working in front of you. And yes, sometimes you do need to address the specific elements of it, but that whole person is a person together.”* **OT 2**

Only one of the participants suggested that MH training should be incorporated within mandatory training for all healthcare professionals.

*“Nurses, doctors, you know everybody that does mandatory training. This is what depression is. This is what anxiety is. This is what you know, schizophrenia is. This is what psychosis is. And you know, cause, I think it’s very much left to the individual clinician to decide what is important to them and what you know, it’s sort of external training. And I think it should be mandatory. I think it should be included for everyone.”* **Physio 4**

#### The role of supervision

The majority of participants expressed the importance of engaging with a designated supervisor/mentor who is a senior colleague with previous experience or a psychologist/psychiatrist.

*“I think one of the things that’s probably really important is supervision. Having that space to be able to reflect on your caseload, and when those people have come up with mental health problems or you’re concerned about that is having someone to go to who can supervise appropriately and steer people in the right place. And in my other team I’m supervised by a psychologist, which is so, you know, it’s brilliant.”* **OT 3**

Any training courses or supervision would be acceptably delivered in person or face-to-face.

*“Maybe a module… maybe 6 to 8 days of learning and then I think it would be good to have follow up sessions…maybe once or twice a year… and then do that within your team at work to have some sort of learning and share information.”* **Physio 7**

#### Time and cost

Concerns about costs and time to train during working hours were raised by seven participants. Participants attended training if their institutions covered fees and provided the time. However, three participants were not averse to self-funding and making time to advance their MH expertise.

*“To pay, that’s another factor. If my trust pays. I would love it. If I had to pay, you know. […] I think it really depends on how passionate you are about mental health on top of what you’re doing. […] Personally, I’d love it if it was in a reasonable amount.”* **OT 1**

*“yeah, definitely. The time is the barrier, at the moment. So something has got to give in order to do that.“* **Physio 1**

## Discussion

This study aimed to explore the experiences of UK AHPs, particularly physiotherapists and OTs, working with patients with MSK and co-existing MH conditions and to understand views on improving MSK services. The findings indicate that AHPs recognise the impact of co-existing MH conditions on the management trajectory of MSK conditions and that integration of psychological skills in parallel to the current biomechanical approach is both favourable and warranted. However, not all expressed an interest towards delivering psychological interventions and challenges to achieving effective integration were equally cited, which were consistent with the existing literature [19, 20, 29, 44]. These challenges go beyond the need for additional training and knowledge acquisition and may relate to departmental readiness and sub-optimal care pathways at a higher organisational level.

### Openness to provide mental health support

Irrespective of the openness to provide MH support expressed by most AHPs, lack of confidence and perceived risk of violating the scope of practice repeatedly contributed this overarching theme, which is consistent with the existing physiotherapy evidence base [20, 21]. Although training and advanced knowledge are important factors for enhancing confidence [45], interestingly a lack of confidence and feeling underprepared was also reported by all participants who had previously engaged in mental health training, including OTs who are already taught at the undergraduate level and have designated roles in promoting mental health [31, 46]. Similar findings have also been reported for OTs within the community setting [29]. These observations suggest that training alone may be inadequate for facilitating effective integration of biomechanical and psychological skills within the MSK setting. For example, even with adequate training, there is still a lack of sufficient detail within clinical practice guidelines, to guide AHPs regarding the appropriate level of involvement needed for the management of psychological aspects [47]. This was evidenced by participants who raised concerns about the risk of violating their scope of practice. Future research exploring the views of psychologists towards the delivery of MH interventions by AHP might shed more light on this.

Since the integration of psychological skills within MSK practice might be acceptable to some AHPs and not all, this highlights opportunities to upskill some MSK practitioners in mental health, as part of initiatives to develop Advanced Practice Practitioners (APPs), who ‘work with a high degree of autonomy, use complex decision-making within multidisciplinary teams, and work across the health and social care system to enable patient centred care’ [48]. Similar Advanced Clinical Practitioner roles are also available to OTs [49], even though uptake of these positions aren’t as high as physiotherapists [50]. There is also some evidence to suggest that APPs can be a cost-effective alternative to usual medical or psychological care [51].

### Challenges

Previous systematic reviews identified time constraints, lack of training, role clarity and confidence as barriers to integrating psychological skills within physiotherapy practice [19, 20]. In addition to these previously cited challenges, the current study identified an overwhelming acknowledgement towards the challenges associated with delivering psychological interventions within the boundaries of the current organisational structure of the health service. Constrained appointment schedules do not match the complex needs of the MSK population, which are rising due to multimorbidity [3] and increasing prevalence of co-existing mental health conditions [5, 6, 52]. The evolving needs of the MSK population could therefore benefit from more flexible appointment schedules, particularly in outpatient settings where appointments are notoriously controlled, with limited room for adjustment. This is of particular importance since evidence suggests that outpatient facilities were the most common setting for evaluating mental health interventions in patients with chronic conditions [18]. These observations suggest there is a need to focus on understanding how the delivery of psychological interventions can be feasibly implemented from the perspective of NHS managers. A further challenge identified by AHPs related to the experience of situations where there was an expectation to treat patients’ physical symptoms, rather than their psychological symptoms. Although mental health awareness has improved over the last decade [53], it is possible that information about the association between physical and mental co-morbidities isn’t widely available within the public domain and that there is limited public awareness about the roles of non-psychologists (such as AHPs) for the management of psychological symptoms. Targeted public health promotion initiatives and campaigns may be required to improve understanding.

### Training

This overarching theme acknowledged previous training and focused on the need for further training, where participants discussed perspectives on implementation. Despite some of the cited challenges in this study, such as mental health stigma, and challenges within the clinical environment discussed in overarching theme two, half of the participants reported having previously engaged with ‘additional’ mental health training, defined as training/courses beyond their undergraduate curriculum and/or mandatory training. This included training on skills to identify/screen mental health conditions and apply non-pharmacological management strategies, such as motivational interviewing. All three OTs were amongst the five AHPs who reported previous engagement with mental health training, which may be reflective of their more established roles in mental health and designated mental health modules included within their undergraduate curriculum [31, 46]. Furthermore, 9/10 participants were in more senior positions (band 6 and above), which may account for why half of the group may have previously received training. This is not necessarily representative of more junior AHPs [54].

Despite the high uptake of additional training amongst AHPs in more senior positions, there was a mismatch between the previous uptake of training and perceived confidence/ knowledge, as all participants expressed a need for further training and a lack of confidence. This suggests that training in psychological skills should be tailored to the requirements of AHPs working in the MSK setting. For example, there may be a need to focus training on the identification and management of common mental health conditions that are prevalent within the MSK population, such as anxiety and depression [10, 55]. However, irrespective of the training available to AHPs and their willingness to engage in MH management, current care pathways are not appropriately structured to facilitate the integration of these skills as highlighted under the theme of ‘work related pressures’. These need to be considered in parallel by managers if effective physical and psychological integration can be operationalised in future MSK settings.

### Strengths and limitations

To the authors knowledge, this is the first study to explore the experiences of AHPs (physiotherapists and OTs) who manage patients with co-existing anxiety and depression within an MSK setting, as previous studies have explored this topic within a specific profession [19, 20, 56]. Furthermore, considerable heterogeneity amongst the existing evidence base is evident, such as findings reported from surveys [21, 25], studies that have focused on elite athletes [23, 24], or qualitative interviews involving physiotherapists from outside the UK [26–28]. OTs and physiotherapists work collaboratively in MKS settings and the MDT biopsychosocial rehabilitation has been associated with positive outcomes [33]. Therefore, these eligibility criteria make these findings unique to this type of practice. However, the authors acknowledge that there was a small sample of participants included in this study, particularly with representation from OTs (n = 3). While statistics indicate that physiotherapists account for the largest professional group amongst AHPs [57], patients who seek care within an MSK setting present with multiple physical conditions that are responsive to physical rehabilitation (such as exercise). It is possible that physiotherapists have a greater exposure to MH conditions in light of evidence that suggests that the prevalence of MH conditions are higher amongst people with physical conditions [14]. As such, the study may have attracted a greater interest from the physiotherapy workforce, especially in light of their limited use of psychological interventions for the management of MSK conditions.

Data saturation was also reached after 10 participants, and continuous on-going familiarisation and coding of the data was conducted to aid this process [43]. We recommend that future studies building on this topic use more rigorous methods for sample size determination [58, 59], particularly within the OT profession. Participants were recruited through social media, which could have introduced a volunteer bias. This refers to the potential for volunteers to have certain pre-determined views ready to convey [60]. However, in contrast, social media facilitates the ability to reach masses of potential participants across the country to reduce the effect of selecting a convenience sample at a specific department or location within the United Kingdom [61].

The interviews were conducted by two student researchers. However, data collection and analysis were supervised by two experienced qualitative researchers. The authors also acknowledge that the views and opinions expressed in this study predominantly relate to more senior professions (i.e., band 6 and above) and may therefore not be reflective of more junior practitioners.

## Conclusion

This is the first qualitative study to consider the experiences and views of physiotherapists and OTs towards the necessary support required to optimise the management of patients with MSK and co-existing mental health conditions. It was clear that the MSK AHPs frequently encounter patients who required MH support. These complex patients are challenging to manage and further MH training is both welcomed and necessary. However, training might not be sufficient to facilitate the effective integration of physical and psychological management per se, and there is a need to consider organisational adaptations, such as allocating longer appointment times for more complex cases. The openness of some AHPs to upskill in MH in response to the evolving needs of the MSK population also raises opportunities to develop Advanced Practice Practitioners in MH.

We recommend future research considers the views and experiences of more junior AHPs, as well as exploring the views and perceptions of psychologists towards the delivery of psychological interventions by AHPs. Additionally, there is a need to explore the perspectives of MSK managers towards the feasibility of adapting current organisational pathways to allow for greater flexibility to optimise the support of people with more complex needs using a combined MDT and biopsychosocial approach.

### Electronic supplementary material

Below is the link to the electronic supplementary material.

Appendix 1

## Data Availability

The datasets used and/or analysed during the current study are available from the corresponding author on reasonable request.

## List of abbreviations

AHP: Allied health care professional
APP: Advanced Practice Practitioner
CBT: Cognitive Behavioural Therapy
ICSP: Interactive Chartered Society of Physiotherapy
ITA: Inductive thematic analysis
MDT: Multi-disciplinary team
MSK: Musculoskeletal
NHS: National Health Service
OT: Occupational Therapist
PIS: Participant information sheet
SCI: Spinal Cord Injury
UK: United Kingdom
