# Correction: Allied health professionals’ experiences and views towards improving musculoskeletal services in the UK for patients with musculoskeletal and co-existing mental health conditions: a qualitative study

**DOI:** 10.1186/s12891-024-07515-w

**Published:** 2024-05-23

**Authors:** Rokhsaneh Tehrany, Dana Maki, Maria J C Teixeira, Tanya Chumak, Christine Hoerz

**Affiliations:** 1 Department of Health Sciences, College of Health, Medicine and Life Sciences, Brunel University, London, UK; 2 Alanzoor Physiotherapy & Rehabilitation Complex, Manama, Kingdom of Bahrain; 3 Therapies Department, Royal National Orthopaedic Hospital NHS Trust, London, UK; 4 Department of Orthopaedic and Musculoskeletal Science, University College London, London, UK; 5 Nursing Research Department, Royal National Orthopaedic Hospital NHS Trust, London, UK; 6 London South Bank University, London, UK; 7 Nuffield Health Oxford, The Manor Hospital, Oxford, UK

**Correction:** ***BMC Musculoskelet Disord*** **25, 207 (2024)**

10.1186/s12891-023-06878-w

Following publication of the original article [1], the order of the first two authors (Dana Maki and Rokhsaneh Tehrany) has been swapped in the author list.

Old order of authors:

Dana Maki^1,2^, Rokhsaneh Tehrany^3,4^*, Maria J C Teixeira^5,6,7^, Tanya Chumak^1^ and Christine Hoerz^1^.

Correct order of authors:

Rokhsaneh Tehrany^3,4^*, Dana Maki^1,2^, Maria J C Teixeira^5,6,7^, Tanya Chumak^1^ and Christine Hoerz^1^.

The original article [1] has been corrected.

## References

[1] Tehrany R, Maki D, Teixeira MJC (2024). Allied health professionals’ experiences and views towards improving musculoskeletal services in the UK for patients with musculoskeletal and co-existing mental health conditions: a qualitative study. BMC Musculoskelet Disord.

