# Supplementary material for: Allied health professionals’ experiences and views towards improving musculoskeletal services in the UK for patients with musculoskeletal and co-existing mental health conditions: a qualitative study
Source: BMC Musculoskelet Disord. 2024 Mar 7;25:207. doi: 10.1186/s12891-023-06878-w (PMC10918939; doi:10.1186/s12891-023-06878-w)
Supplement: Supplementary file 1 — Appendix 1 [file 12891_2023_6878_MOESM1_ESM.docx]

Appendix 1

| Question | Prompts |
| --- | --- |
| 1. Talk to us about your experience working with patients in MSK settings with MH conditions | - What are your personal experiences working with patients that have co-existing MH conditions? - Have you treated them before? - Can you give examples of MH conditions you have seen? - How do you deal with patients who have both an MSK condition and a co-existing MH condition? - Can you describe your preparedness to work with patients who have MH conditions in MSK settings? - Can you describe the role of the physio/OT who encounters patients with MSK and co-existing MH conditions? |
| 1. Talk to us about any previous MH training that you have had, if any? | If yes:   - Can you describe the content, style, frequency. - What went well? - What could be better? - Was it useful?   If no:   - What are your views on doing training? - What would you like to learn, and how? - Will it change your practice? |
| 1. What are your thoughts on MH training for AHPs? | - Can you tell me about your thoughts own experiences? - What are your thoughts on pre-registration training? - What are your thoughts on post-registration training? - Any other comments. |
| * Would you like to tell us anything else about working with patients with both MSK and MH conditions? | - Can you elaborate? - Can you give examples? |
